# Circ_0000527 promotes the progression of retinoblastoma by regulating miR-646/LRP6 axis

**DOI:** 10.1186/s12935-020-01396-4

**Published:** 2020-07-10

**Authors:** Li Zhang, Jie Wu, Yujun Li, Yanxia Jiang, Lili Wang, Yunqing Chen, Yalin Lv, Yuwei Zou, Xiaoyan Ding

**Affiliations:** 1 Department of Pathology, The Affiliated Hospital of Qingdao University, Qingdao, 266003 Shandong China; 2 Department of Dermatology, The Affiliated Hospital of Qingdao University, No 16, Jiangsu Road, South District, Qingdao, 266003 Shandong China

**Keywords:** RB, Circ_0000527, miR-646, LRP6, ceRNA

## Abstract

**Background:**

Researches validate that circular RNAs (circRNAs) are dysregulated in a variety of malignancies and play an important role in regulating the malignant phenotype of tumor cells. Nevertheless, the role of circ_0000527 in retinoblastoma (RB) and its regulatory mechanisms remain largely unknown.

**Methods:**

Real-time PCR (RT-PCR) was used to detect circ_0000527 and miR-646 expression in RB tissues and cells. The LRP6 expression in RB cells was detected by Western blot. The relationship between circ_0000527 expression and the clinicopathological parameters of RB patients was analyzed. Cell proliferation, apoptosis and metastasis were monitored by cell counting kit-8 (CCK-8), flow cytometry, and Transwell assay. The dual-luciferase reporter gene assay and RIP assay were employed to verify the targeting relationship between circ_0000527 and miR-646, miR-646 and LRP6.

**Results:**

Circ_0000527 was highly expressed in both RB and RB cell lines, whose high expression level and degree of differentiation were significantly correlated with the increase in cTNM staging level. Overexpression of circ_0000527 contributed to RB cell proliferation, migration, invasion and suppressed cell apoptosis, while knocking down circ_0000527 inhibited the above malignant biological behavior. The underlying mechanism suggested that functioning as a endogenous competitive RNA, circ_0000527 directly targeted miR-646 and positively regulated LRP6 expression.

**Conclusion:**

Circ_0000527 enhances the proliferation and metastasis of RB cells by modulating the miR-646/LRP6 axis.

## Background

Retinoblastoma (RB), the most common intraocular malignancy, usually occurs in childhood [1], which accounts for 2–4% of all malignancies in children under 5 years of age and can easily cause vision loss or eye loss or even death [2].

Unlike other linear RNAs, circular RNAs (circRNAs) lack the 3′ and 5′ ends, which protects it from the degradation by RNA exonuclease, so they can be more stably present in tissues and cells [3, 4]. Accumulating experiments indicate that circRNAs can function as biomarkers for cancer diagnosis and potential therapeutic targets [5]. For instance, circ_0000745 enhances cervical cancer (CC) cell proliferation, migration, invasion and epithelial-mesenchymal transition (EMT) [6]; circ_0058124 facilitates the progression of papillary thyroid carcinoma by modulating the NOTCH3/GATAD2A axis [7]; circ-SERPINE2 up-regulates the expression of YWHAZ and promotes the progression of gastric cancer [8]. However, the researches on expression function and mechanism of circRNA in RB are relatively rare.

MicroRNAs (miRNAs), small non-coding RNAs (ncRNAs) embraceing 19–25 nucleotides, inhibit gene expression by binding to the 3′ untranslated region (3′-UTR) of the target mRNA [9]. Increasing experiments find that miRNAs participate in many aspects of cancer biology, such as cell proliferation, differentiation, apoptosis and metastasis [10]. For instance, in renal clear cell carcinoma, miR-205-5p suppresses VEGFA expression and the PI3K/Akt/mTOR signaling pathway to function as a tumor suppressor [11]; miR-204-5p represses the proliferation and invasion of esophageal squamous cell carcinoma cells by suppressing IL-11 [12]. MiR-646 is considered as a tumor suppressor in several cancers. For example, miR-646 inhibits gastric cancer cell proliferation and EMT-induced metastasis by targeting and suppressing FOXK1 expression [13]. Nonetheless, the expression and function of miR-646 in RB remains largely undefined.

The low-density lipoprotein receptor-related protein 6 (LRP6) is a member of the low-density lipoprotein (LDL) receptor gene family [14]. The protein encoded by this gene acts as a receptor for Wnt or as a co-receptor with Frizzled, involved in the classical Wnt/β-catenin signaling pathway [14]. By cascading with the Wnt/β-catenin signaling, this gene plays a prominent role in regulating cell differentiation, proliferation, migration, as well as the deterioration of diverse cancer types, including RB [15, 16]. Furthermore, it has been reported that the expression level of LRP6 is modulated by a plurality of miRNAs, such as miR-202-3p and miR-126-3p [17, 18]. However, the mechanism which is responsible for the dysregulation of LRP6 expression in RB has not been fully elucidated.

Interestingly, with bioinformatics analysis, we noticed that LRP6 was a potential target gene of miR-646. Meanwhile, miR-646 was a potential target of circ_0000527. In this study, we investigated the expression pattern of circ_0000527 expression in RB. Furthermore, we explored whether circ_0000527 was involved in the progression of RB via regulating the miR-646 and LRP6.

## Materials and methods

### Tissues collection

45 cases of RB patients and their adjacent tissues were harvested in the Department of Pathology, Affiliated Hospital of Qingdao University. The clinical and pathological features of each patient were collected postoperatively. The study equipped with written informed consent from the guardians of the patients and this study was endorsed by the requirements of the Ethics Committee of the Affiliated Hospital of Qingdao University.

### Cell culture and transfection

The RB cell lines (WERI-RB-1, SO-RB50, Y79, and RB355 cells), human retinal pigment epithelial cells (ARPE-19) and kidney epithelial cell HEK293T cells were purchased from the American Type Culture Center (ATCC, Rockville, MD, USA). All cells were cultured in Dulbecco’s Modified Eagle’s Medium (DMEM, Thermo Fisher Scientific, Shanghai, China) containing 10% fetal bovine serum (FBS; Gibco, Grand Island, NY, USA) and 1% penicillin/streptomycin (Invitrogen, Carlsbad, CA, USA), and the cells were maintained in a 5% CO_2_ humidified incubator at 37 °C. The cells were passaged at intervals of 2 to 3 days, and the cells in the logarithmic growth phase were selected for experiments.

After being trypsinized with trypsin, the cells were then seeded in 24-well plates and cultured in an incubator to continue the culture for 24 h. The circular transcript expression vector possesses two elements termed as the front circular and the back circular frame which were specially designed containing inverted repeat sequences flank. The full-length cDNA of circ_0000527 was amplified in RB cells, and was cloned into the specific vector between two frames. Small interfering RNA (siRNA) against circ_0000527 (si-circ_0000527), the pcDNA 3.1 and the si-NC were purchased from Genechem (Shanghai, China). miR-646 mimics, miR-646 inhibitor, negative control miR-con were purchased from Ribobio (Guangzhou, China), and then they were transfected into WERI-RB-1 cells and Y79 cells according to Lipofectamine™ 3000 (Invitrogen, Carlsbad, CA, USA) transfection reagent instructions. The transfection efficiency was detected 48 h later.

### Quantitative real-time polymerase chain reaction (qRT-PCR)

According to the manufacturer’s instructions, the total RNA was extracted from tissues and cells using TRIzol reagent (Thermo Scientific Hyclone, Utah, USA). After detecting the purity of total RNA with a spectrophotometer, 3 μg of total RNA was used as a template, and the RNA was reversely transcribed into cDNA employing the reverse transcription kit (Thermo Scientific Hyclone, Utah, USA). qRT-PCR detection was subsequently performed using BestarTM qPCR MasterMix. GAPDH and U6 were considered as internal parameters, and the 2^−ΔΔCT^ method was employed to calculate the relative expression levels of the molecules. The primer sequences were available from Thermo Fisher Scientific (Shanghai, China), and more details could be found in Table 1.

**Table 1:** Primer sequences used for qRT-PCR

circ_0000527	F: TCAACCAGGTGGATGTGTGG
	R: GACTCGCTCGAGAGGGGTTA
miR-646	F: ACACTCCAGCTGGGAAGCAGCTGCCTC
	R: CTCAACTGTGCTGCATTAGTTAGCTCAGA
U6	F: CGCTTCGGCAGCACATATACTA
	R: CGCTTCACGAATTTGCGTGTCA
LRP6	F: AGG CACTTACTTCCCTGCAA
	R: GGGCACAGGTTCTGAATCAT
GAPDH	F: CGGATTTGGTCGTATTGGG
	R: CTGGAAGATGGTGATGGGATT

*F* forward, *R* reverse, *RT* reverse transcription

### The cell counting kit (CCK-8) assay

CCK-8 was adopted to examine the effects of circ_0000527 and si-circ_0000527 or miR-646 mimics and miR-646 inhibitors on RB cell viability. RB cells were seeded into 96-well plates at a density of 1 × 10^3^ cells per well. The cells were cultured at 37 °C for 24, 48, and 72 h, and incubated with CCK-8 solution (10 μL) (Dojindo, Kumamoto, Japan) for 10 min, and then the absorbance in each well was measured at 450 nm using a microplate reader (Bio-Rad, Hercules, CA, USA).

### Flow cytometry

Y79 and WERI-RB-1 cells in the logarithmic growth phase were collected, and single-cell suspension was prepared and fixed in 70% ethanol at 4 °C overnight. Afterwards, the cells were resuspended in binding buffer and the concentration was modulated to 1 × 10^4^/ml. The cells were stained according to the proportion of 10 μL Annexin V-fluorescein isothiocyanate (Annexin V-FITC) and 5 μL propidium iodide (PI) kit (BD Biosciences, San Diego, CA, USA) per 1 ml cell suspension and incubated in dark at room temperature for 15 min, followed by the addiditon of 400 μL binding buffer. After that, flow cytometry (BD Biosciences, San Jose, CA,USA) was performed within 1 h to analyze the cell apoptosis.

### Transwell migration and invasion assays

Migration and invasion were assessed using a Transwell chamber (Costar, Cambridge, MA, USA) without Matrigel (for migration analysis) and with Matrigel (for invasion assay), respectively. RB cell suspension (2 × 10^4^ cells in 200 μL serum-free medium) was added to the upper compartment and the medium supplemented with 20% FBS (500 μL) was added to the lower compartment. After cells were cultured at 37 °C for 24 h, the cells passing through the membrane were fixed with 4% paraformaldehyde, stained with crystal violet, and counted.

### Western blot

The total protein of each group was extracted using RIPA lysis buffer (Beijing solarbio science & technology co., Ltd, Beijing, China) according to the manufacturer’s instructions. Equal amounts of protein from each group of cells were subjected to SDS-PAGE electrophoresis and then transferred to a polyvinylidene fluoride (PVDF) membrane. After being blocked for 1 h at room temperature in 5% fat-free milk, the membrane was incubated with specific primary antibody at 4 °C overnight. The primary antibody was obtained from Abcam (Shanghai, China): Anti-LRP6 (Abeam, ab134146, 1:500). Then the membrane was incubated with an horse radishperoxidase (HRP)-labeled secondary antibody (Abcam, ab216773, 1:5000) at room temperature for 1 h. After the procedure of wash, the membrane was exposed with ECL chromogenic reagent (Millipore, Bedford, MA, USA), and then was exposed to film to observe the bands.

### RNA immunoprecipitation (RIP) assay

The EZMagna RIP kit (Merck, Darmstadt, Germany) was used for the RIP experiment in accordance with the manufacturer’s instructions. Anti-Argonaute 2 (AGO2) or anti-IgG antibody was coupled to magnetic beads. The cells were transfected with miR-646 or control miRNA, before RIP lysis buffer was utilized to lyse the cells, after which the lysate was incubated with magnetic beads for 6 h at 4 °C. In order to remove the protein, the magnetic beads were rinsed and incubated with the protease K buffer. Next, RNA was immunoprepcipitated, and reverse transcription was performed with Prime-Script RT Master Mix (TaKaRa, Dalian, China). Finally, the expression of circ_0000527 was analyzed by qRT-PCR.

### Dual luciferase reporter assay

TargetScan (http://www.targetscan.org) was used to predict potential binding sites. Dual luciferase reporter assay was employed to detect the binding relationship between circ_0000527 and miR-646, miR-646 and the 3′UTR of LRP6. HEK293T cells were cultured in 12-well plates and co-transfected with vectors containing wild-type or mutant circ_0000527/3′UTR of LRP6 and miR-646 mimics or control miRNA. Firefly and Renilla luciferase activities were measured 48 h after the transfection employing the dual luciferase reporter system (Promega, Madison, WI, USA). Luciferase activity was calculated as the ratio of firefly luciferase intensity to Renilla luciferase intensity.

### Statistical analysis

Data analysis was performed using SPSS 22.0. All experimental data were expressed as mean ± SD, and differences between the two groups were compared using the *t*-test. In this study, *P* < 0.05 was considered as statistically significant.

## Result

### High expression of circ_0000527 in RB tissues and cells and its clinical significance

**Fig. 1:**
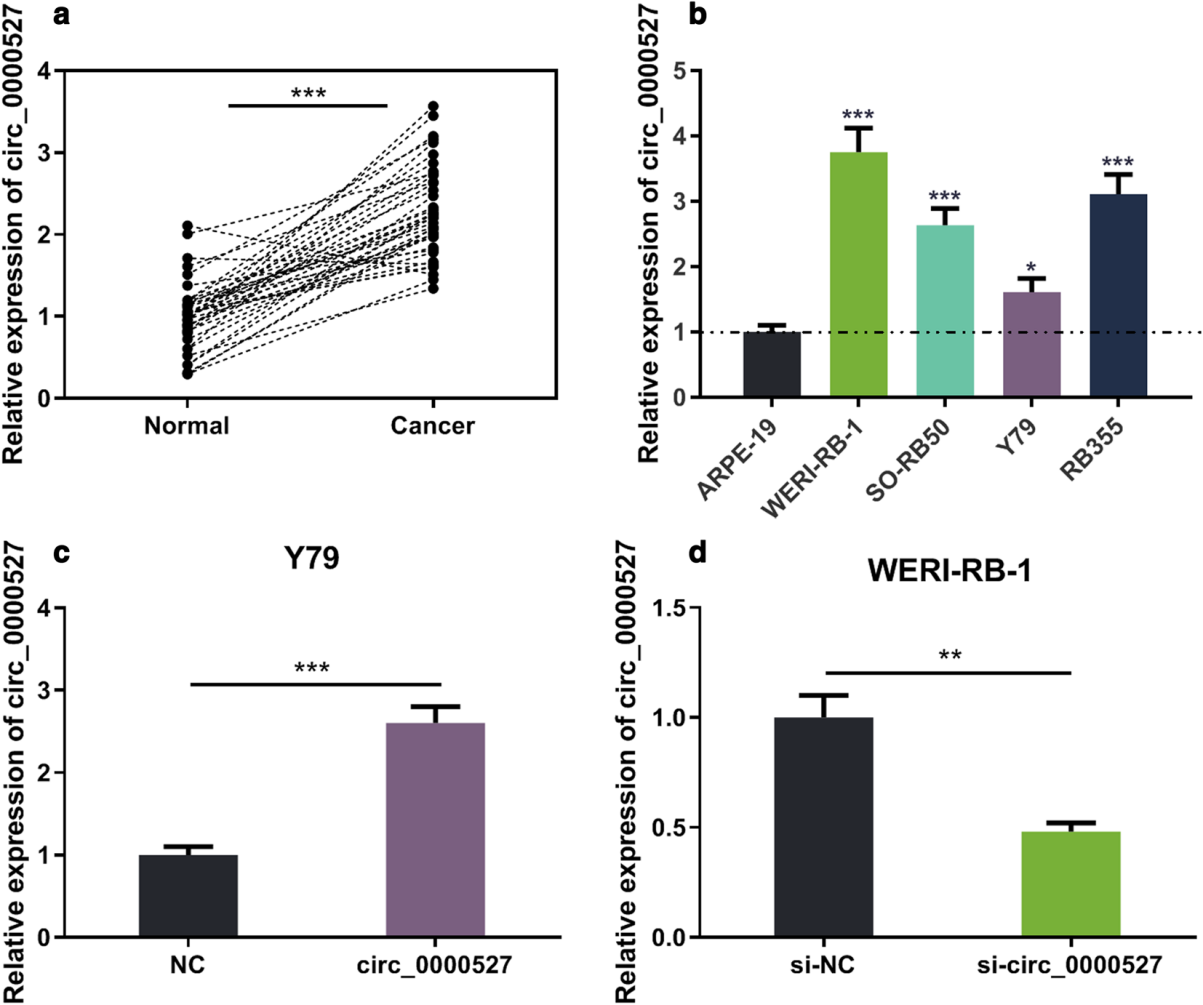
circ_0000527 expression was detected in RB tissues and cell lines. **a** The expression of cric_0000527 in 45 cases of paired RB and paracancerous tissues was detected by qRT-PCR. **b** The expression of cric_0000527 in retinal pigment epithelial cells (ARPE-19) and RB cells was detected by qRT-PCR. **c** The expression level of cric_0000527 was detected by qRT-PCR after transfecting the overexpressing cric_0000527 plasmid into Y79 cells. **d** After transfecting cric_0000527 targeting siRNA into WERI-RB-1 cells, the expression level of cric_0000527 was detected by qRT-PCR. *P < 0.05, **P < 0.01, and ***P < 0.001

To explore the expression characteristics of circ_0000527 in RB tissues and cells, qRT-PCR was performed. The results authenticated that circ_0000527 expression in RB tissues was significantly higher than that in adjacent tissues (Fig. 1a). In addition, compared with retinal pigment epithelial cells ARPE-19 cells, circ_0000527 expression in RB cells (WERI-RB-1, SO-RB50, Y79 and RB355 cell lines) was significantly increased (Fig. 1b). Likewise, by analyzing the relationship between the expression level of circ_0000527 and the clinicopathological parameters of RB patients, we found that the high expression level of circ_0000527 was significantly correlated with the degree of differentiation and the increase of cTNM staging level (Table 2). These studies suggested that circ_0000527 played an oncogenic role in RB.

**Table 2:** Correlations between circ_0000527 and clinical characteristics in RB patients

Pathological indicators	Number of patients	circ_0000527 expression		*P* value
		High	Low	
All cases	25	14	11	
Age (years)				
≥ 5	4	1	3	0.173
< 5	21	13	8	
Gender				
Male	12	6	6	0.561
Female	13	8	5	
Tumor enucleated location				
Right	13	8	5	0.561
Left	12	6	6	
Differentiation				
Well and moderate	15	6	9	0.048*
Poor	10	8	2	
T classification				
T1 + T2	15	7	8	0.250
T3 + T4	10	7	3	
N classification				
N0	11	4	7	0.080
N1 + N2	14	10	4	
cTNM stage				
I + II	6	1	5	0.026*
III + IV	19	13	6	
Largest tumor base (mm)				
≥ 15	9	7	2	0.100
< 15	16	7	9	

*P < 0.05

### circ_0000527 enhanced RB cell proliferation, migration and invasion and suppressed the apoptosis

**Fig. 2:**
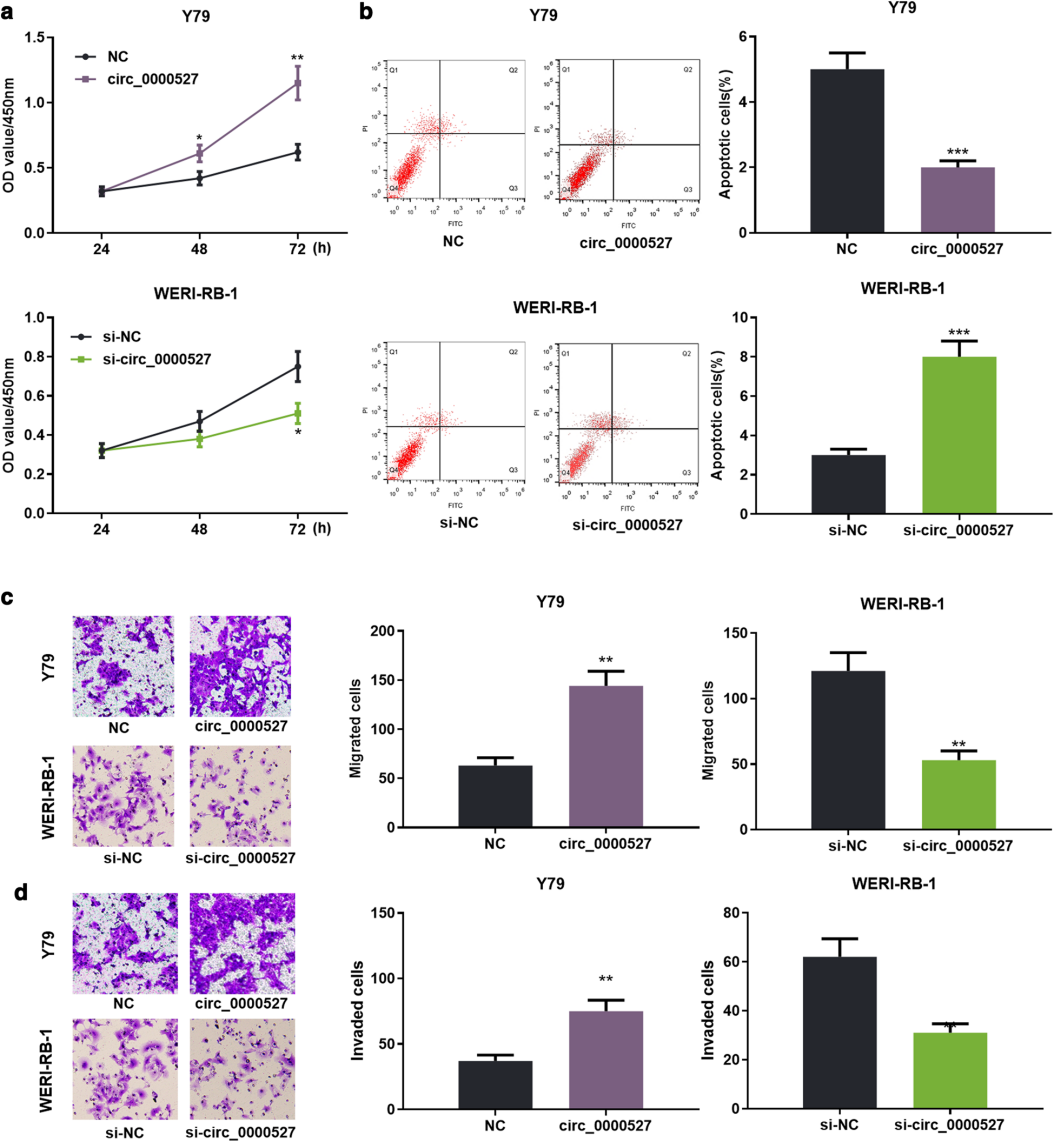
circ_0000527 promoted RB cell proliferation, migration and invasion and inhibited cell apoptosis. **a** RB cell proliferation was detected by CCK-8. **b** The proportion of apoptosis of RB cells was detected by flow cytometry. **c**, **d** Cell migration and invasion were detected by Transwell assay. *P < 0.05, **P < 0.01, and ***P < 0.001

To further probe the role of circ_0000527 in RB, we transfected circ_0000527 plasmid and siRNA targeting circ_0000527 into Y79 and WERI-RB-1 cells, respectively. The transfection was confirmed by qRT-PCR (Fig. 1c, d). After the cell models were successfully constructed, cell proliferation and apoptosis were detected by CCK-8 and flow cytometry, respectively, the data of which manifested that overexpression of circ_0000527 notably enhanced Y79 cell proliferation and suppressed apoptosis compared to the control group (Fig. 2a, b). Transwell assay confirmed that overexpression of circ_0000527 promoted cell migration (Fig. 2c) and invasion (Fig. 2d) compared to the control group, while knocking down circ_0000527 inhibited cell proliferation, migration and invasion, and enhanced cell apoptosis (Fig. 2a–d). These results suggested that circ_0000527 accelerated RB cell progression.

### Targeting relationship between circ_0000527 and miR-646

**Fig. 3:**
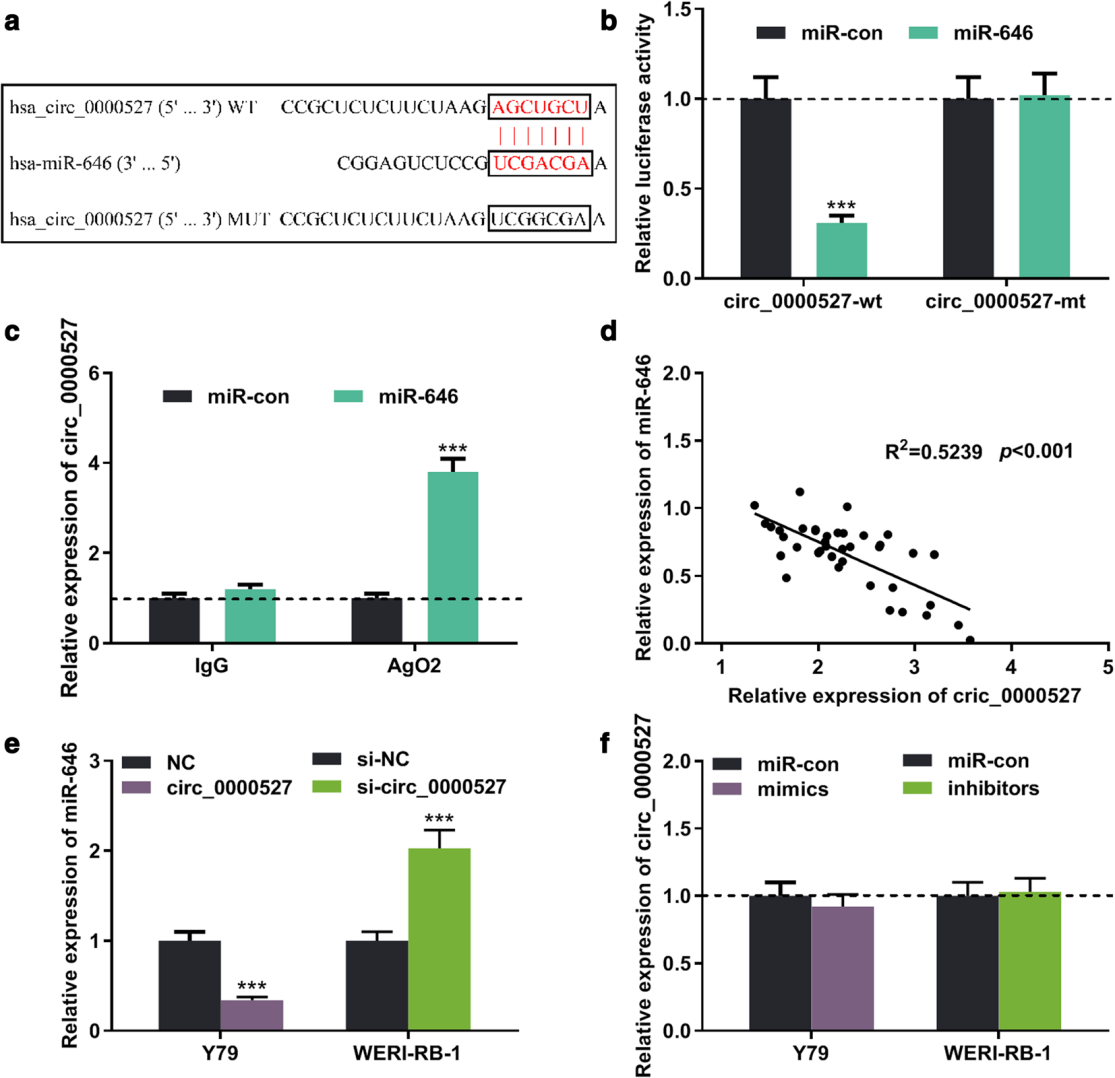
circ_0000527 adsorbed miR-646. a The binding site between cric_0000527 and miR-646. **b** The binding relationship between cric_0000527 and miR-646 was verified by luciferase activity assay in 293T cells. **c** The interaction between cric_0000527 and miR-646 was detected by the RIP assay in 293T cells. **d** The correlation between cric_0000527 and miR-646 expression in RB samples was analyzed by Pearson analysis. **e** The expression of miR-646 was detected by qRT-PCR after up-regulating or down-regulating cric_0000527 in RB cells. **f** The expression of cric_0000527 was detected by qRT-PCR after up-regulating or down-regulating miR-646. ***P < 0.001

Through the StarBase database 3.0 (http://starbase.sysu.edu.cn/panCancer.php), we found that a potential binding site existed between circ_0000527 and miR-646 (Fig. 3a). To verify whether circ_0000527 absorbed miR-646, dual luciferase reporter assay was carried out. The results implied that the miR-646 mimics markedly reduced the luciferase activity of the wild-type circ_0000527 reporter compared to the control group, whereas the luciferase activity of the mutant circ_0000527 reporter was not significantly altered by miR-646 mimics (Fig. 3b). Furthermore, RIP assay confirmed that circ_0000527 and miR-646 were enriched in Ago2-containing microribonucleoproteins compared to control IgG (Fig. 3c). Based on the Pearson correlation analysis, we found a negative correlation between the expression of cric_0000527 and miR-646 in RB samples (Fig. 3d). Additionally, we found that the upregulation of circ_0000527 significantly suppressed the expression of miR-646, and knockdown of circ_0000527 enhanced the expression of miR-646 in RB cell lines (Fig. 3e). Moreover, there was no significant change in the expression of circ_0000527 after up-regulation or inhibition of miR-646 (Fig. 3f). In summary, circ_0000527 was an upstream molecule that could adsorb miR-646 and negatively regulate its expression.

### miR-646 inhibited the malignant phenotypes of RB cells

**Fig. 4:**
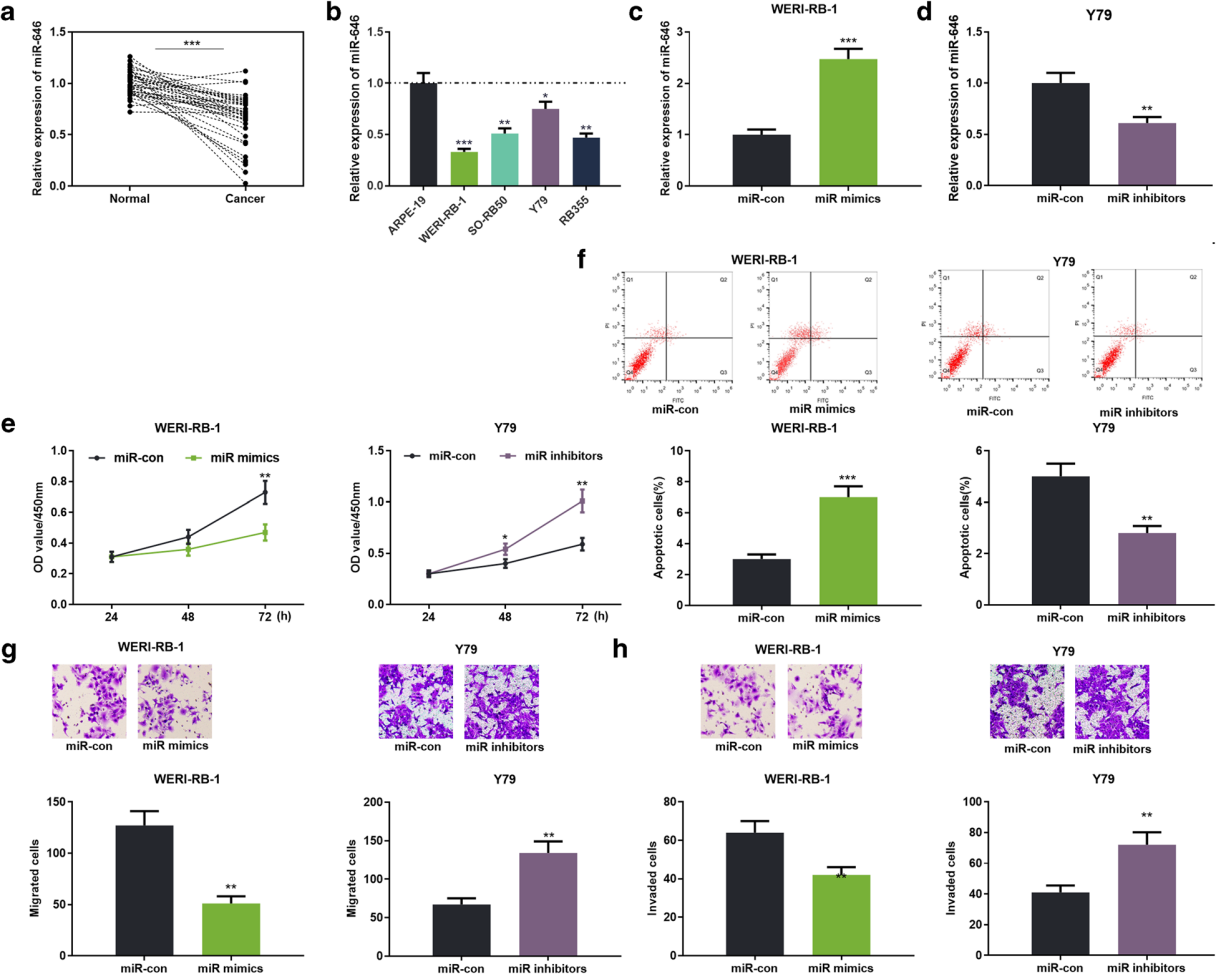
miR-646 functioned as a tumor suppressor in RB. **a** The expression of miR-646 in 45 cases of paired RB and paracancerous tissues was detected by qRT-PCR. **b** The expression of miR-646 in retinal pigment epithelial cells (ARPE-19) and RB cells was detected by qRT-PCR. **c**, **d** The expression level of miR-646 was detected by qRT-PCR after RB cells were transfected with miR-646 mimics and inhibitors. **e** RB proliferation was detected by the CCK-8. **f** Flow cytometry was used to detect apoptosis of RB cells. **g**, **h** Transwell assay was used to detect RB cell migration and invasion. ***P < 0.001, and **P < 0.01

The data mentioned above suggested that miR-646 acted as a downstream molecule of circ_0000527. To investigate the expression and role of miR-646 in RB, qRT-PCR was employed to detect the expression of miR-646 in RB tissues and cells. The results revealed that miR-646 expression was remarkably decreased in RB cancer tissues compared with paracancerous tissues (Fig. 4a); consistently, miR-646 expression was notably decreased in RB cells compared with ARPE-19 cells (Fig. 4b). Then, the miR-646 mimics and the miR-646 inhibitor were transfected into WERI-RB-1 cells and Y79 cells, respectively. The model for high expression and low expression of miR-646 was successfully constructed and verified by qRT-PCR (Fig. 4c, d). CCK-8, flow cytometry, and transwell assay confirmed that overexpression of miR-646 inhibited cell proliferation, migration, and invasion, and enhanced cell apoptosis. On the contrast, inhibition of miR-646 enhanced cell proliferation, migration and invasion, and repressed cell apoptosis (Fig. 4e–h). These results revealed that miR-646 exerted a tumor-suppressive effect on RB.

### circ_0000527/miR-646 axis modulated RB cell proliferation, apoptosis, migration and invasion

**Fig. 5:**
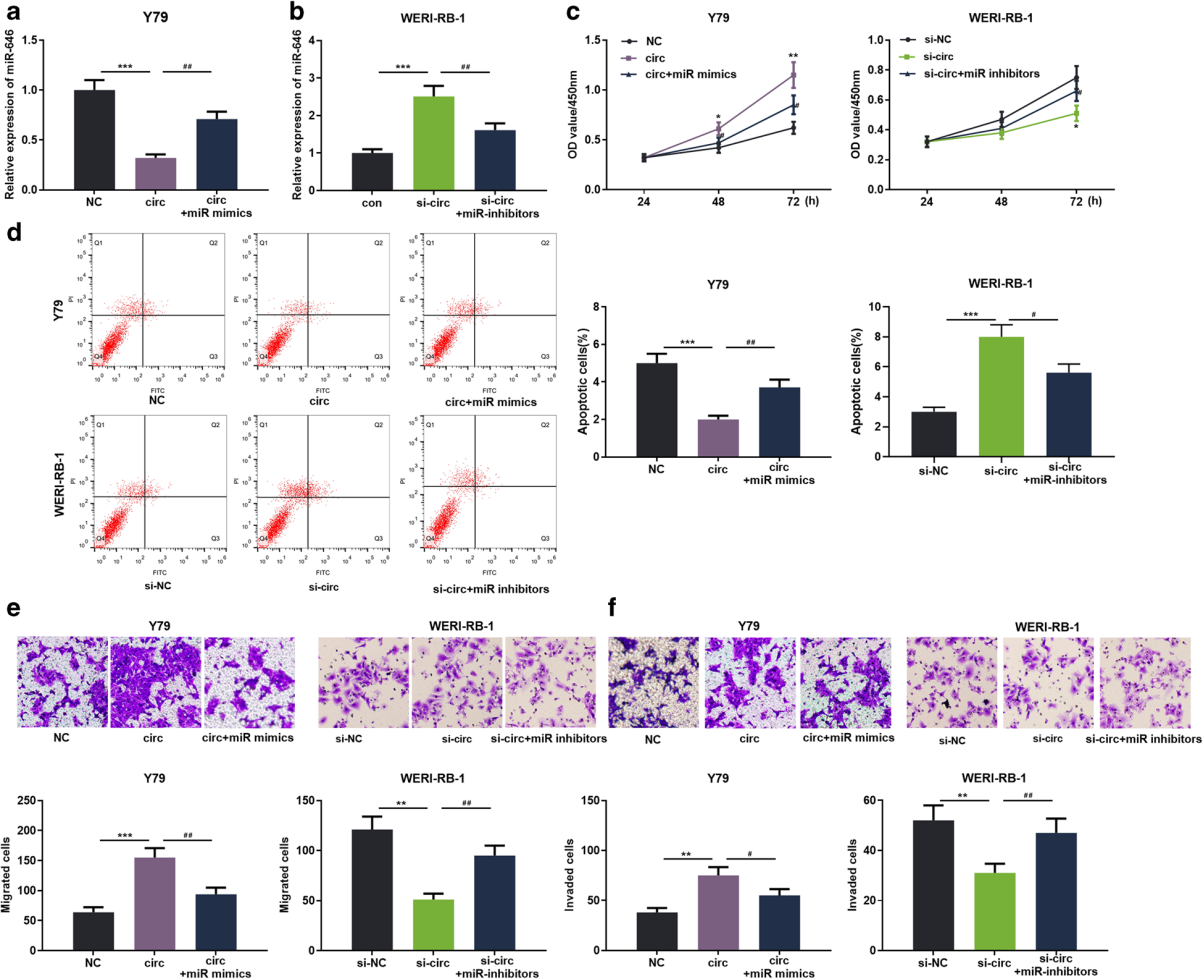
circ_0000527/miR-646 axis regulated RB cell proliferation, apoptosis, migration and invasion. **a**, **b** Transfection efficiency was detected by qRT-PCR. **c** The proliferation was detected by the CCK-8 method. **d** Flow cytometry was used to detect apoptosis of RB cells. **e**, **f** Transwell assay was used to detect RB cell migration and invasion. *P < 0.05, **P < 0.01, ***P < 0.001, NC vs. circ_0000527 or si-NC vs. si-NC. ^#^*P* < 0.05, ^##^*P* < 0.01, circ_0000527 vs. circ_0000527 + miR-646 or si-circ_0000527 vs. si-circ_0000527 + miR-646 inhibitors

In order to explore the function of the circ_0000527/miR-646 axis in regulating the biological behaviors of RB cells, miR-646 mimics and circ_0000527 overexpressing plasimds were co-transfected into Y79 cells, and the miR-646 inhibitor and circ_0000527 siRNA were co-transfected into WERI-RB-1 cells. The transfection was validated by qRT-PCR (Fig. 5a). It was confirmed that co-transfection of miR-646 mimics reversed the effects of circ_0000527 overexpression on RB cells; similarly, co-transfection of miR-646 inhibitors reversed the effects of circ_0000527 knockdown (Fig. 5b–f). These studies indicated that the circ_0000527/miR-646 axis was engaged in RB progression.

### circ_0000527/miR-646 axis regulated the expression of LRP6

**Fig. 6:**
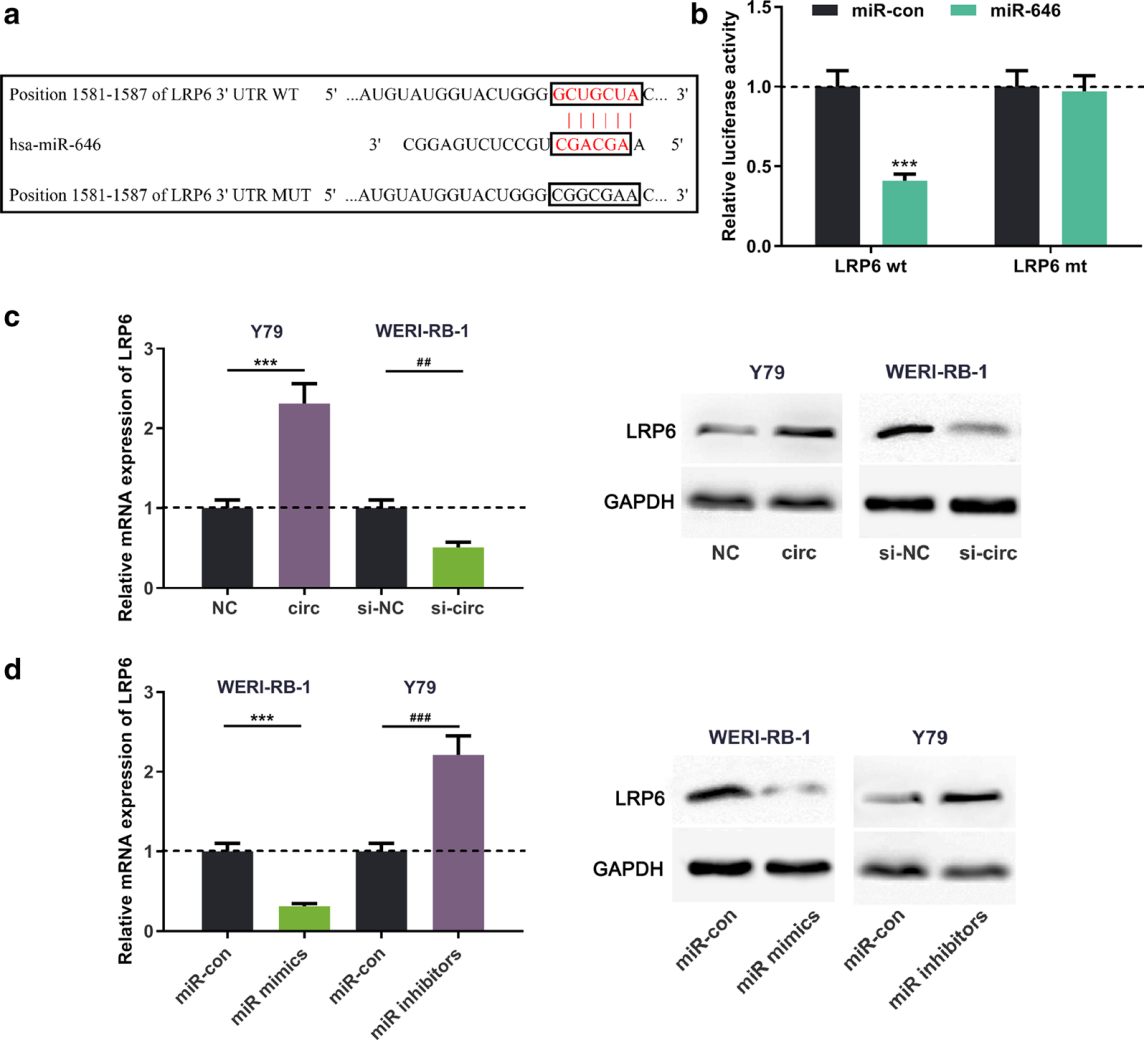
Regulatory effect of circ_0000527/miR-646 axis on the expression of LRP6. **a** The binding site between miR-646 and the 3′UTR of LRP6. **b** The binding relationship between miR-646 and 3′UTR of LRP6 was verified by luciferase activity assay. **c** The expression of LRP6 was detected by qRT-PCR and Western blot after up-regulation or down-regulation of cric_0000527, respectively. **d** The expression of LRP6 was detected by qRT-PCR and Western blot after up-regulation or inhibition of miR-646, respectively. ***P < 0.001, and **P < 0.01

To further explore the downstream molecules of miR-646, we found through the TargetScan database that there was a potential base-complementary binding site between miR-646 and the 3′UTR of LRP6 (Fig. 6a). To verify whether miR-646 binded to LRP6, dual luciferase reporter assay was carried out and the results showed that the miR-646 mimics remarkably decreased the luciferase activity of the wild-type LRP6 3′UTR -WT reporter compared to the control, but did not change that of the mutant reporter (Fig. 6b). In addition, qRT-PCR and Western blot assay verified that the expression of LRP6 mRNA and the protein was increased by circ_0000527 overexpression or miR-646 inhibition in RB cells, while knocking down circ_0000527 or upregulating miR-646 caused a decrease in LRP6 expression (Fig. 6c, d). Besides, the expression of circ_0000527 was verified to be positively correlated with that of LRP6 in RB and paracancerous tissues (Additional file 1: Figure S1). These results implied that LRP6 was negatively regulated by miR-646 and was positively regulated by circ_0000527.

## Discussion

Recently, ncRNAs including circRNA have increasingly become a hot topic in the field of cancer research [19, 20]. In-depth study of the role of circRNA in RB and its regulatory mechanisms is of substantial value to provide a theoretical basis for elucidating the mechanism of RB progression [21]. In this study, we found for the first time that circ_0000527 expression was remarkably up-regulated in RB tissues and cell lines. Furthermore, its high expression level is significantly associated with adverse clinical features of RB patients. In terms of mechanism, circ_0000527 accelerated the progression of RB by adsorbing miR-646 and activating LRP6 expression.

CircRNAs, derived from reverse splicing or exon skipping of pre-mRNAs [5], is stable due to its special ring structure and resistant to exonuclease [22]. The role of circRNA in tumorigenesis and cancer progression has attracted widespread attention. Functionally, circRNA participates in the formation of tumors, regulating malignant biological behaviors of cancer cells such as proliferation, apoptosis and metastasis. It is reported that circ_0001649 suppresses RB cell proliferation and apoptosis by inhibiting AKT/mTOR signaling pathway, and its low expression level indicates poor prognosis in patients [23]; circ_ODC1 positively regulates SKP2 and promotes RB cell proliferation [24]. In this study, we found for the first time that the expression level of circ_0000527 in RB tissues and cells was significantly increased, and its high expression was significantly correlated with the lower the degree of differentiation and increased cTNM staging. These results indicated that circ_0000527 was expected to be a predictor of poor prognosis of RB patients. In addition, the proliferation, migration and invasion of the cells were remarkably promoted by circ_0000527 overexpression, while the proportion of apoptosis of cells was remarkably reduced. Conversely, knocking down circ_0000527 exerted the opposite effect. The above results suggested that circ_0000527 was involved in promoting the progression of RB and demonstrated that it had an important role in RB.

MiRNAs are also reported to be engaged in the regulation of the tumorigenesis and progression of a variety of human tumors [25, 26]. MiR-646 is regarded as a tumor suppressor in many human cancers. For instance, in lung cancer, miR-646 suppresses cell proliferation and metastasis by negatively regulating the EGFR/Akt pathway [27]; in colorectal cancer, miR-646 represses NOB1 to inhibit cancer progression [28]. MiR-646 suppresses clear cell renal carcinoma cell metastasis by downregulating nin one binding protein [29]. MiR-646 is involved in inhibiting proliferation and metastasis of non-small cell lung cancer by binding to FGF2 and CCND2 [30]. These studies suggested that miR-646 plays an anticancer role in a variety of tumors. In this study, we found a significant decrease in miR-646 expression in RB tissues and cell lines. In vitro assays confirmed that miR-646 overexpression remarkably repressed RB cell proliferation and metastasis and enhanced cell apoptosis, whereas the inhibition of miR-646 significantly promoted the aforementioned malignant biological behaviors. It should be noted that previous researches show that circRNAs regulate gene expression by adsorbing miRNAs [31]. In view of the distinct roles of circ_0000527 and miR-646 in RB progression, we made a hypothesis that there was such a relationship between them. As we expected, we identified a potential binding site between miR-646 and circ_0000527, and we found that circ_0000527 could negatively regulate miR-646. These data implied that in RB, the functions of circ_0000527 were partly mediated by miR-646.

LRP6 is a transmembrane protein involved in receptor-mediated endocytosis of lipoproteins and protein ligands [32]. This protein forms a complex with transmembrane receptor members of the Frizzled family and acts as a co-receptor in the classical Wnt/β-catenin signaling cascade [33]. It is reported that LRP6 is up-regulated in various tumors, including colon cancer, breast cancer, as well as RB [16, 34, 35]. In this study, we found that the upregulation of miR-646 and knockdown of circ_0000527 induced a decrease in LRP6 expression in RB cells. On the other hand, inhibition of miR-646 or overexpression of circ_0000527 enhances LRP6 expression. Therefore, we concluded that the circ_0000527/miR-646 axis contributed to the dysregulation of LRP6 in RB.

## Conclusion

In conclusion, our study presents a novel body of experimental data in support of circ_0000527 as a tumor-promoting circRNA in RB. We find for the first time the high expression level of circ_0000527 in RB tissues and cells, whose high expression is related to RB patients’ adverse clinicopathological parameters. In terms of mechanism, we confirm that circ_0000527 promotes cell proliferation, migration, and invasion by modulating the miR-646/LRP6 axis, which is expected to elucidate the mechanism of RB progression. Therefore, circ_0000527/miR-646/LRP6 axis can serve as a promising and effective therapeutic target for RB patients. However, the sample of this research study is limited to a single center, and and larger sample size is needed in the future to further confirm this conclusion.

## Supplementary information

**Additional file 1: Figure S1.** The correlations between the expression levels of circ_0000527 and LRP6. (A) The expression level of circ_0000527 was positively correlated with that of LRP6 in RB. (B) The expression level of circ_0000527 was positively correlated with that of LRP6 in paracancerous tissues.

## Data Availability

The data used to support the findings of this study were available from the corresponding author upon request.

## Abbreviations

circRNAs: Circular RNAs
RB: Retinoblastoma
RT-PCR: Real-time PCR
CCK-8: Cell counting kit-8
miRNAs: MicroRNAs
3′-UTR: 3′ untranslated region
LRP6: Low-density lipoprotein receptor-related protein 6
DMEM: Dulbecco’s Modified Eagle’s Medium
FBS: Fetal bovine serum
siRNA: Small interfering RNA
PVDF: Polyvinylidene fluoride
AGO2: Anti-argonaute 2
