# Supplementary figures and images for: Circ_0000527 promotes the progression of retinoblastoma by regulating miR-646/LRP6 axis
Source: Cancer Cell Int. 2020 Jul 10;20:301. doi: 10.1186/s12935-020-01396-4 (PMC7350616; doi:10.1186/s12935-020-01396-4)

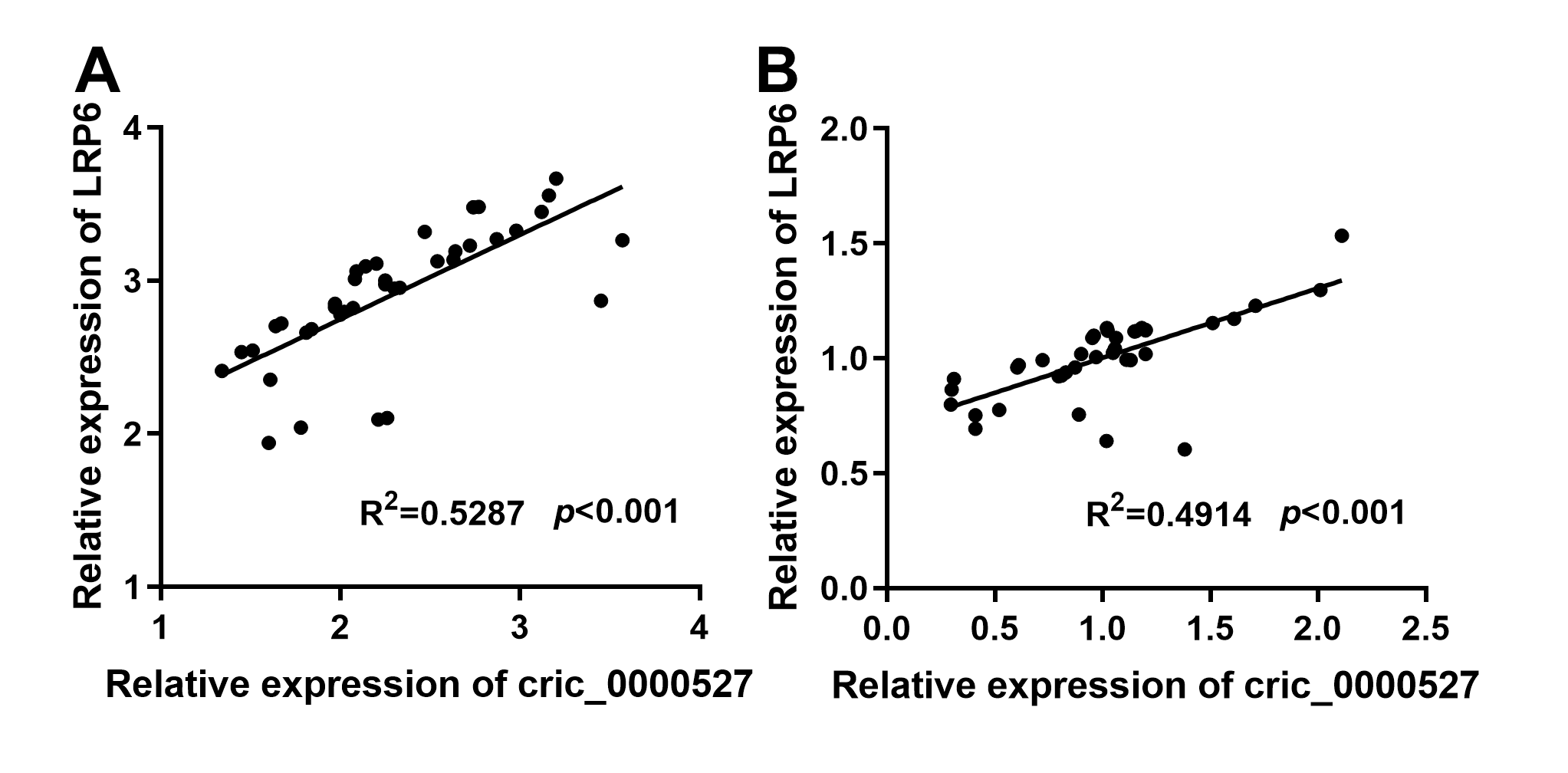

Supplement: Supplementary file 1 — Additional file 1: Figure S1. The correlations between the expression levels of circ_0000527 and LRP6. (A) The expression level of circ_0000527 was positively correlated with that of LRP6 in RB. (B) The expression level of circ_0000527 was positively correlated with that of LRP6 in paracancerous tissues. [file 12935_2020_1396_MOESM1_ESM.tif]
